# Exploring simulation as a teaching pedagogy for male undergraduate nursing students: A Qualitative Study in the United Arab Emirates

**DOI:** 10.12688/f1000research.167350.2

**Published:** 2025-10-06

**Authors:** Jacqueline Maria Dias, Mini Sara Abraham, Muhammad Arsyad Subu, Nabeel Al Yateem, Fatma Reefat Ahmed, Raliya Abdourahman Abdillahi, Noof Jamal Saleh, Samira Mohamed Ali, Kowsar Abshir Hashi, Richard Mottershead

**Affiliations:** 1 Department of Nursing, College of Health Sciences, University of Sharjah, Sharjah, United Arab Emirates; 2 Institute of Health Sciences, Binawan University, Jakarta, Indonesia; 3 Sakina, Behavioural Science Institute, SEHA, Al Ain, United Arab Emirates; 4 College of Nursing, University of Baghdad, Baghdad, Iraq

**Keywords:** Simulation, male students, baccalaureate, nursing, pedagogy, education, United Arab Emirates.

## Abstract

**Background:**

The nursing profession is increasingly appealing to male students at the undergraduate level. Society’s demand for nurses, particularly male nurses, is rising; nevertheless, the availability of clinical placements for nursing students is insufficient to meet this demand. Simulation has significantly advanced as an educational method for training healthcare personnel. Simulation makes replicating real-life events and honing skills like clinical decision-making and communication. Using simulation as an adjunct or alternative to clinical practice is now under discussion. Simulation is a resource-intensive pedagogical approach, necessitating careful consideration of its organization and implementation to maximize efficiency without undermining learning outcomes.

**Objective:**

This study aimed to explore simulation as a teaching pedagogy for male undergraduate nursing students in the United Arab Emirates.

**Method:**

This study adopted a descriptive qualitative approach. The purposive sampling method recruited 43 male students from nursing programs in the University of Sharjah, United Arab Emirates, to participate in the individual interviews. In addition, two focus group discussions were conducted on an online meeting platform. Data analysis was done using thematic analysis.

**Results:**

In this study, we identified four major themes: 1) Students’ perceptions of simulation, 2) Preparation for realistic situations, 3) Feeling of anxiety and stress, and 4) Theory and practice abilities gaps.

**Conclusion:**

The study findings indicate that male nursing students received the overall simulation experience well. The study highlights the importance of skilled and encouraging simulation instructors who can provide participants with valuable guidance and feedback for more individualized teaching. Study results provide important new information about how simulation-based learning could improve nursing education and further studies in this field. The findings shed light on the problems that male nursing students have been dealing with for years and highlight efforts to make changes as soon as feasible. It may be helpful to revise the simulation to provide more opportunities for independent problem-solving skills and to incorporate more real-life factors and variables into the scenario. Additionally, developing more individualized approaches to building self-confidence may be helpful, such as providing personalized feedback and guidance based on each student’s strengths and weaknesses.

## Introduction

Increased knowledge, improved satisfaction with skill performance, and improved critical thinking and self-confidence in the students’ skill set were the goals of the educational outcomes. Nursing education includes both theoretical and practical instruction to educate students in nursing. Developing knowledge and clinical skills depends on the time allotted to teaching and clinical practice and the content of both. Simulation is how we attempt to attain outcomes that closely resemble clinical practice ( Khan et al., 2022). It is a nursing instructional strategy that simulates patient care experiences by re-enacting scenarios or real-life circumstances. It offers nursing students and working nurses a secure and regulated setting to develop their clinical expertise. Simulation can help students gain knowledge, hone their clinical reasoning abilities, and become capable of caring for patients and their families in a complicated healthcare setting ( Aebersold, 2018). Over time, its technology has advanced significantly, allowing for replicating complex patient scenarios and conditions through high-fidelity manikins and simulated environments ( Herrera-Aliaga & Estrada, 2022).

In nursing education, simulation allows students to practice in a safe setting. Simulations are valuable because they give students real-world experience and allow them to reflect on their learning while playing various roles ( Haddeland et al., 2021). Simulations teach students to share responsibility, encourage one another, give constructive criticism, communicate clearly, and be honest about negative experiences ( Jeffries, 2022). In higher education, simulation-based learning substantially contributes to student-centered, collaborative, active, and interprofessional learning ( Glavin, 2009).

Various sources emphasized the value of simulation as a training method that offers a secure, authentic setting to improve learning and develop nursing abilities, along with how these skills can affect critical thinking, judgment, problem-solving, and self-confidence. Additionally, the experiences of male nursing students exposed to lab training using high-fidelity simulation has been reported earlier by the same authors. Furthermore, simulation has proven to be an effective tool for instruction in nursing as an interactive teaching method ( Khan et al. 2022). A study had the same result ( Smith, Jordan, & Li, 2022). According to other research, clinical internships and simulation exercises are closely related examples of how practical teaching and learning form the foundation of nursing education ( Dias et al., 2023; Ahmed et al., 2025). The capacity to identify symptoms, conduct evaluations, and take necessary action was displayed for others to observe and evaluate ( Peachey, 2021). Simulation made clinical judgment on subscales, including observing, interpreting, responding, and reflecting ( Ayed et al., 2022). However, prior research has concentrated on how students respond to negative emotions when learning through simulation ( Ko & Choi, 2020). However, when it comes to encouraging learning, positive emotions work better than negative ones ( Keskitalo & Ruokamo, 2021). The students experienced positive and negative emotions during the simulation ( Salo et al., 2025). Teachers should therefore be mindful of the stressors in the simulation scenario, such as sensitivity in interpersonal interactions, unfamiliarity with new learning methods, the weight of being observed, and dread of evaluation ( Ko & Choi, 2020).

More male nursing students are enrolling in educational institutions in the United Arab Emirates ( Subu et al., 2022). However, the number of real-world training opportunities available for these students is not keeping up with the increasing demand. Simulation as a teaching pedagogy is essential to integrate nursing students into the field and provide them with the necessary skills ( Rattani et al., 2020). The maternal health course has presented several difficulties as male students register in nursing programs. In addition, because of cultural restrictions that prevent them from entering the maternity hospital, nursing faculty find it challenging to locate suitable practice opportunities in obstetrics for male baccalaureate nursing students due to the conservative Arab culture. Students were given simulated experiences to gain the skills required for the course, such as maternal health, to overcome these cultural barriers to clinical learning and guarantee that they met the maternal course competencies.

### Study purpose

In the Gulf region, few clinical settings can give bachelor male nursing students thorough, profound, and high-quality hands-on learning experiences in maternity care, where patients’ needs are complex and hospital stays are brief, limiting male students’ opportunities. Simulation is utilized to improve students’ clinical fundamental competencies while navigating through significant issues due to societal and cultural concerns. Understanding the unique experiences that male nursing students face during simulation is important. This study aimed to explore simulation as a teaching pedagogy for male undergraduate nursing students. The findings will benefit clinical practice in identifying nursing students’ experiences in the healthcare setting and improve teaching strategies to help shape and enhance future curricula.

## Methods

### Design

This study used a descriptive qualitative approach to explore simulation as a teaching pedagogy for male undergraduate nursing students in the United Arab Emirates. Qualitative research methods are regularly used in healthcare professions research and nursing research. Qualitative studies were employed to enhance comprehension and elucidate human experience. In qualitative research, data is gathered through interviews and is methodically collected and analyzed. The interview questions can be found in Table 1. It investigates the meanings of social phenomena in which people perceive circumstances ( Grossoehme, 2014), focusing on examining how people understand their experiences and the meaning they give them.

**Table 1.** Interview questions.

Question number	Interview question	Probe
1	**How would you describe your overall experience during the maternity practicum labs?**	*What stood out the most to you?*
2	**What were your feelings and thoughts on the first day of your maternity practicum?**	*Were you nervous, excited, or unsure?*
3	**How did your feelings change by the last day of the practicum?**	*What had improved or become more comfortable?*
4	**Can you share a positive experience you had during your maternity practicum?**	*What made it a good experience?*
5	**Can you describe a negative or challenging experience during the practicum?**	*How did you handle it?*
6	**What was the most difficult part of your experience in the maternity lab or clinical setting?**	*Skills, communication, environment, etc.*
7	**As this was your first exposure to maternity care, what specific fears or anxieties did you experience?**	*Were any of these related to gender, confidence, or knowledge?*
8	**Did participating in the maternity practicum affect your daily life or your mental/emotional health? If so, how?**	N/A
9	**What specific challenges did you face as a male student in the maternity practicum setting?**	*Interaction with patients, acceptance by staff, cultural barriers, etc.*
10	**What advice would you give to a second-year male student who will soon enter the maternity practicum?**	*What do you wish you had known beforehand?*
11	**How were you able to apply maternity theory learned in class during your practicum or simulation sessions?**	*Can you give specific examples?*
12	**Would you consider working in the maternity department in the future? Why or why not?**	*Did your practicum influence this decision?*
13	**How did simulation activities in the sim labs prepare you for real-life maternity clinical experiences?**	*What scenarios or skills were most helpful?*

**Settings and participants**

This study was conducted in the nursing program at the University of Sharjah, United Arab Emirates. The study population was undergraduate nursing students. The purposive sampling method recruited thirty-two male students from nursing programs to participate in the individual face-to-face interviews. According to Charmaz (2006), 25 participants in a qualitative study are more than enough to reach data saturation. In addition, two focus group discussions were conducted on an online meeting platform (Microsoft Teams). The first focus group involved six participants from the fourth year, and the second had five participants from the third year. In this study, there are 43 student participants in total. This study’s inclusion criteria are male nursing students at the University of Sharjah who completed the maternity practicum courses in year three. Students also must speak English and be willing to participate in the research. The exclusion criteria were the students who did not complete the simulation component. Bridging students and transfer students were also excluded.

### Data collection

This study employed semi-structured interviews as the primary data collection method. Richards and Morse (2007) assert that a semi-structured interview is a recognized technique for data collection in qualitative research, facilitating constructive interactions with participants and enabling them to express their thoughts, experiences, attitudes, and beliefs. After we received approval from the REC University of Sharjah, we contacted each potential male participant via email or phone. We approached potential participant personally and face-to-face. Prior to participation, all students received a clear explanation of the study’s objectives, procedures, and their rights as participants. Written informed consent was obtained from all participants. We interviewed each participant at their college at the University of Sharjah. Study participants who consented to partake were invited, and we elucidated the study’s objective along with the schedule and location of each interview. All interviews were conducted with the participants’ consent in the nursing program at the University of Sharjah. Individual interviews were audiotaped using a tape recorder with participants’ consent, and each interview lasted 40 to 50 minutes. As a triangulation, we conducted two focus group discussions on an online meeting platform (Microsoft Teams) to ensure consistency and obtain more participant feedback. Triangulation employs many approaches or data sources in qualitative research to thoroughly understand phenomena ( Patton, 1999). Triangulation is a qualitative research approach for validating findings by synthesizing data from many sources ( Carter et al., 2014). The first focus group involved six participants from the fourth year, and the second had five participants from the third year. Each discussion lasted approximately 35 minutes. One experienced supervisor (JD, the leading researcher) organized the FGD sessions with two groups.

### Data analysis

**Figure 1.**
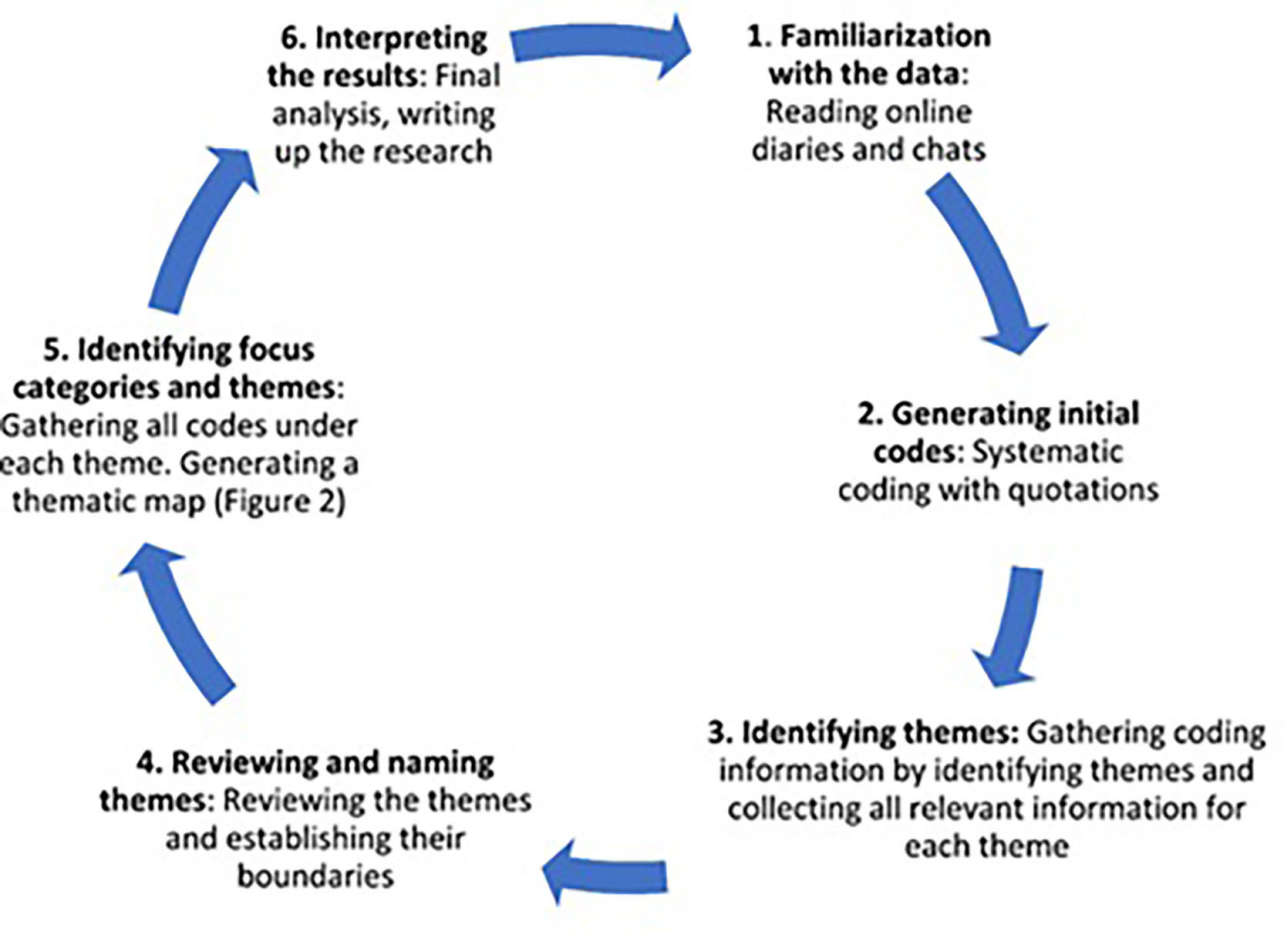
Phases of thematic analysis ( Braun & Clarke, 2006).

This study adopted a thematic analysis and adhered to the six data analysis phases described by Braun and Clarke (2006)( Figure 1). In this qualitative study, we employed NVivo 12 as our data management tool, which assists us in coding and identifying themes—thematic analysis. These tool helps us interpret interviews and focus groups, uncover patterns, and derive insights in identifying themes. The thematic analysis objective is to identify data patterns pertinent to the study’s purpose. Phase one (phase familiarization) involved verbatim transcriptions of every interview. This study conducted all the FGDs and interviews in English.

At this stage, notes were taken as a beginning point. We created initial codes in step two by performing line-by-line coding, marking, and assigning codes to essential information from the raw data. In the third phase, known as ‘searching for themes,’ all codes are input into a different spreadsheet, where all authors assess and discuss them to find and classify similar codes into themes. In step four, we review the themes to determine their validity in terms of internal homogeneity. Coding disagreements in qualitative research can happen when different coders assign varying labels to the same data, reflecting different interpretations or ambiguities. This study addresses coding disagreements through systematic procedures, including detailed discussions where coders justify their choices, consult established guidelines, and seek input from third parties to maintain transparency and clarity. During our analysis, the authors meet to review all coding and reach consensus. Regular meetings are held throughout the process to review the data and implement any necessary adjustments. To ensure the themes relate to the data and make the themes and their connections more straightforward, we reviewed all the data again in phase five (defining and naming themes). Our findings are presented in a document generated in the last phase (phase six, creating the report). Braun and Clarke (2006) state that the writing moved from data summaries to analytical tales.

**Ethical consideration**

The University of Sharjah’s Research Ethics Committee (REC) REC-23-02-27-02-S approved this research project.

Participants received a thorough explanation of the research prior to the interview. Every student participating in the study was aware of its purpose and methodology. Prior to each interview, we gave each participant signed informed consent. They received guarantees regarding the study’s secrecy and anonymity. Students who participated in the study were informed that it was entirely voluntary and that they might leave at any moment. Furthermore, their grades were unaffected by their involvement in this study. To ensure that every participant felt at ease and secure, we chose interview settings for this study that could offer seclusion and silence. Furthermore, none of the participants in this study were paid directly for their time.

**Study rigor**

A participant’s private information disclosed to a researcher is considered confidential. There are restrictions on when and how it can be shared with a third party. Without their explicit consent, study participants were guaranteed that their data would remain anonymous. Keeping a person’s name apart from their involvement in a study is known as anonymity, and no names were used in this study. Each participant was assigned A random alpha-numeric code so that no one could associate a transcription with a specific participant (P1 for Participant 1). A password-protected private computer was used to download the digitally recorded interviews. Researchers should always be the only ones handling the interview material, including the recordings and transcriptions. All private information and documents were secured in the University of Sharjah’s chief researcher’s office. Data were stored for five years after the study ended, and then the material was destroyed (tapes were demagnetized and transcriptions were shredded).

## Results

In this study, forty-three male students, aged between 19 and 23, participated. All verbatim transcripts from the interviews were thoroughly read and extensively discussed with all authors. Two focus group interviews were conducted in a virtual meeting for the study. Six students participated in the first FGD in the fourth year, while the second FGD included five participants from the third year. The interviews’ audio recordings were fully transcribed, and transcripts of the interviews were checked against written summaries of the contributions made by each interviewer. After reviewing the transcripts, we identified four major themes: 1) Students’ perceptions of simulation, 2) Preparation for realistic situations, 3) Feeling of anxiety and stress, and 4) Theory and practice abilities gaps.

### Theme 1: Students’ perceptions of simulation

***Subtheme: Practice in the same way with realistic situations***

The study participants stated that even if they know what must be done, they must get the contact nurse’s permission before acting in real-world practice settings. They practice in the simulation much like in actual clinical settings.

*… I believe it is great that this course is about nursing care. The fact that it makes sense for us has, I believe, been evident throughout the instruction. If the simulations in the training had taken place in the department… It would have been practiced similarly in realistic situations, like in a similar experience. (FGD 1, member 1).*

Similarly, a participant said in an interview that he practices with simulation in the same way as with realistic situations in health settings.

*In my opinion, the course’s connection to nursing care is fantastic. It has become evident throughout the instruction that it makes sense for us. Things would have been different if we had taken a simulation course set in a hospital. Since we mimicked these scenarios, we encountered numerous things that raised our level of knowledge and confidence while we were there [clinical placement]*

*(Interview, participant 17)*

***Subtheme: Improve knowledge and skills***

The male students said the simulation enhanced their understanding of equipment best practices and procedures. Their practical skills were strengthened as they gained confidence and increased awareness of the tools and neonatal care procedures. One student thought back to how, prior to the simulation, he had no idea how to practice using the chart.

*Before the simulation, I had no idea how to use resuscitation equipment or score the baby prior to the physical activity. After arriving here for the simulation, I learned how to operate this equipment. It improved my knowledge and skills… I have no fear of anything related to the realities of caring for a patient (FGD 2, member 3).*

Similar to the statement above, another participant said:

*…. I was nervous to utilize it when I first saw it in a clinical setting, but after practicing with a simulation, I became more comfortable with it. I was also able to use it and successfully perform resuscitation on a neonate when I returned to the clinical area. It will enable us to conduct many more procedures effectively (Interview, participant 26).*

***Subtheme: Increased self-confidence***

The students, who had never done such things before, greatly appreciated learning about clinical procedures and more complex difficulties outside the clinical context. In addition to lowering their anxiety of making mistakes in secure learning settings, the students felt that the simulation increased their confidence, which is essential for clinical practice and professional life.

*… The simulation enhances our confidence and prepares us psychologically and physically for practice. Because the simulation represents the reality of what we do in clinical settings, I felt more confident and gained more experience during the scenario… (FGD 1, member 5).*

The students indicated that simulation exercises improved their confidence and capacity to perform in clinical settings. Because of the simulation, students said they felt more confident and performed better.

*… Because simulation speeds up processes, it has increased my confidence and performance in the clinical setting. I was not confident in my practice before, but going to the simulation sessions, where we practice a lot, has given me more self-assurance (Interview, participant 22).*

***Subtheme: Improve communication skills***

Male students also discussed how they learned that effective communication is essential to managing neonatal emergencies. They believed requesting help during the simulation promoted interprofessional interaction, communication, and teamwork.

*My ability to communicate effectively in the clinical setting has improved through simulation. I am sufficiently knowledgeable to interact with other colleagues while performing my tasks once the situation occurs in the clinical setting. ask for help … (FGD 2, member 2).*

### Theme 2: Preparation for realistic situations

Based on the interview, the male students’ knowledge increased following the simulation course, and their comprehension of women’s difficulties broadened, which will help them in practical circumstances.

*… Yes, excellent. Being a married man, this course taught me a lot about women and pregnancy, and once I am done, I will be able to support my wife throughout her pregnancy (Interview, participant 15).*

*Everybody will have infant issues because their families will always have neonates. Once we are all married, you will apply the knowledge from the maternity course in certain circumstances, particularly in emergency scenarios or real life (FGD 2, member 4).*

***Subtheme: Integrating theory into practice***

Through interactions and criticism with facilitators and other students, the students felt that the simulation session was essential to improving their critical thinking and reasoning skills. This critical thinking and introspection benefited the development of clinical knowledge and abilities.

*I experience… A simulation can help you learn about your weaknesses, how each person engaged in the scenario, and integrate a theory into clinical practice. You are also asked why you did this, which aids with memory recall. Therefore, I had more opportunities to gain theory and knowledge in the clinical practice (FGD 1, member 3).*

Students found simulation essential to integrating and applying their theoretical knowledge to clinical practice. During the simulation, they believed the facilitators improved their knowledge and abilities by encouraging them to think critically and reason more.

*Through observation and logic, simulation has enhanced our understanding and application of any care we offer. Simulation can also enhance our reasoning skills (Interview, participant 29).*

***Subtheme: More practice time in simulation labs***

The students lamented the lack of simulation laboratories throughout the maternity course, making it difficult to do well on the OSCE tests. They would only retain the checklist information without understanding what it meant. The second issue is the lengthy time between practice and the exam.

*I request that the doctor give the students more than one to two labs or more than two weeks to repeat the experience because we knew then, but now we have forgotten. They [instructors] only gave us two labs and the exams, which is insufficient (Interview, participant 29).*

*Even though we had the checklist step by step, they could not fully comprehend it during the OSCE examination. We need more time to practice in the labs so they can be familiar with the procedure, which would help them in the OSCE examination (FGD 1, member 4).*

### Theme 3: Feeling of anxiety and stress

During the simulation, the male students felt anxiety and stress due to cultural and religious factors. Some felt stress and anxiety because other students or instructors would evaluate them.

*Yes, I am stressed and uncertain… the measurement procedures were carried out during the simulation. I also had a fear of touching the babies because we are boys, and that anything could happen to the baby (FGD 1, member 6).*

### Theme 4: Theory and practice abilities gaps

The discrepancy between the knowledge acquired in theoretical training and its application in a clinical context is known as the theory-practice gap. The misalignment between the theoretical portion and the simulation labs caused some participants to feel inept. It inhibited them from applying their skills or acquiring new ones on the clinical days.

*In simulation, my problem is the gap between lab training and the OSCE exam. I did nothing except the assessment tool and went home after that. The theory and practice in the lab, the gap is there… (Interview, participant 18).*

*At the beginning of the course, we only took the basics in the lab, and there was a long gap between clinical labs and my clinical practices, so it was not much of a surprise for me… (FGD 2, member 4).*

## Discussion

In this study, we found that male participants generally had a positive perception of the objectives and information provided during the simulation. These results suggest that the simulation design provided a supportive learning environment, with participants feeling that their needs were recognized and that support was available on time. The male student participants felt the support provided was effective in helping them learn and problem-solve during the simulation. Using simulation as a teaching tool can help by giving students realistic chances to participate in learning activities that call for making their own clinical decisions and seeing the outcomes of their answers in a secure setting ( Newton & Krebs, 2020; Saifan et al., 2021). Faculty can detect knowledge and education gaps through simulation, and students can remark on what happened during the simulation experience ( Ali & Musallam, 2018; Gantt et al., 2018).

Simulation has increased nursing students’ confidence, which was not previously known in the literature ( Malya et al., 2025). According to earlier studies, simulation helped nursing students become more competent and confident by providing them with safe practice ( Khan et al., 2022; Dias et al., 2024a). Given that pre-service education frequently lacks opportunities for practical experience, these findings are novel and pertinent, indicating that simulation can boost students’ confidence in providing first neonatal care ( Khan et al., 2022; Al Gharibi and Arulappan, 2020; Bailey & Emory, 2022). It is crucial to impart information and competence to students during their education ( Kruk et al., 2018). To prepare students to become competent professionals who can improve the quality of neonatal care in settings with a high neonatal mortality rate, it is essential to use simulation to increase their procedural confidence ( Khan et al., 2022; Tjoflåt, Koyo, & Bø, 2021; Dias et al., 2024b). Furthermore, during simulation practice, students’ physiological stress and anxiety levels rise, which may impact their performance in real-world scenarios and simulations ( O’Regan, Ekelund, & Watterson, 2022).

Simulation is important since it prepares for realistic situations. Simulation allows nursing faculty to replicate the clinical environment, immersing students in a nonthreatening, guided learning experience. Our findings indicated that most male students indicated that simulation is a way to prepare for realistic situations in clinical settings. Salo et al. (2025) believe that better instruction and greater student satisfaction in simulation-based learning can result from acknowledging and treating students’ emotions. To achieve the optimal learning results, nursing students must improve their practical competence and be ready to work in relationships with people who have not had exposure to the maternal health setting. The study participants indicated that the simulation experience was supportive and provided feedback. Also, the simulation was well-designed to provide participants with the necessary support and feedback to learn and apply new skills effectively in real situations in clinical settings. According to research, simulation can help students gain clinical reasoning abilities and knowledge while preparing them to provide patient and family care in a complicated healthcare setting ( Aebersold, 2018). Following best practice recommendations is necessary for simulation to effectively replace or supplement traditional clinical experiences ( Newton & Krebs, 2020). Nursing students saw simulation as a helpful way to prepare for and enhance clinical practice ( Furuheim & Lindenskov, 2025).

Research on the emotional experiences of nursing students during simulation has shown that they go through a range of coexisting and fluctuating feelings, such as pleasurable and unpleasant ( Salo et al., 2025; Madsgaard et al., 2022). However, most research has been on how nursing students respond to negative emotions when learning through simulation ( Ko & Choi, 2020). Our results showed that several students experienced stress and anxiety during simulation exercises. Emotions also impact how information is encoded, retrieved, and used to solve problems ( LeBlanc & Posner, 2022). According to Caloca-Amber et al. (2024), emotional control, tension, worry, and self-assurance can impact learning outcomes, knowledge, and abilities. Unpleasant feelings like tension and worry interfere with students’ ability to study ( Salo et al., 2025; Amimaruddin & Ruditaldris, 2022). Proper preparation, including outlining the learning objectives, can increase psychological safety and lessen students’ fear, improving their educational experiences ( Somerville et al., 2023). According to Roh and Jang (2017), students frequently feel stressed during simulations, vulnerable, and at high risk of making mistakes or coming off as insufficient. Effective learning requires addressing this problem, and simulation is essential. The students felt the stress of being watched and assessed by facilitators and observers. According to studies, students experience stress and anxiety when participating in a simulation ( Tosterud et al., 2020). Since emotions greatly influence learning, it is critical to examine nursing students’ feelings during simulation ( Madsgaard et al., 2022). Students can be better prepared for problems in the workplace, and a supportive learning environment can be created by acknowledging and treating their emotions. Training professionals with emotional intelligence can help them become well-prepared ( Ahn et al., 2023). According to He et al. (2024), students’ subjective emotions are crucial in perceiving and engaging with their learning environment. The students’ past experiences and knowledge should be the foundation for this. Through carefully thought-out briefings, the facilitator can lower stress levels by acquainting students with the simulation room, the simulator’s capabilities, and the facilitator’s expected role throughout the simulation. A thorough briefing can avoid misunderstandings during the simulation ( Rattani, 2020). Simulation-based learning can be stressful and challenging, impacting learning ( LeBlanc & Posner, 2022). It elicits strong student emotions ( Behrens et al., 2021). With various professional groups and patients, students take on various responsibilities and engage in collaborative learning ( MacLean et al., 2019). In this situation, group skills and emotional control are crucial. Simulations elicit both positive feelings (like surprise and attention) and negative emotions (like dread and anxiety) in nursing students, according to other studies ( Madsgaard et al., 2022). In a simulated environment, students who experience less anxiety might perform better clinically ( Al-Ghareeb, McKenna, & Cooper, 2019). Positive simulation progress experiences heighten positive emotions like confidence, serenity, and a sense of competence, which are thought to affect learning positively. On the other hand, students who felt like they had failed at simulations experienced negative emotions such as stress, worry, fear, despair, and uneasiness. This made them doubt their ability to perform in the future and was thought to have a detrimental effect on their learning ( Behrens et al., 2021). Positive emotions are generally better at fostering learning than negative ones ( Keskitalo & Ruokamo, 2021). Simulations have emerged as a helpful tool for practicing the application of academic information in real-world skill-use scenarios.

The ability of nursing students to integrate classroom knowledge and apply it to clinical practice must be strengthened by nursing instructors. Thus, the theory-practice gap in education may be closed by repeatedly using simulation games and virtual simulation to approximate theory-based teaching practices ( Dias et al., 2023). In nursing education, the theory-practice gap is a significant issue. Nurse educators frequently find it challenging to incorporate theoretical knowledge into their lessons. Educators or mentors may either not know which theoretical knowledge student educators have learned, or their priorities for teaching are inconsistent with the knowledge learned in university. Commonly employed in the simulation phase, pedagogical techniques like debates, reflections, critical thinking, and reasoning are underutilized in many low-income higher health education institutions ( Bø et al., 2022; Tjoflåt, Koyo & Bø, 2021).

The gap between theory and practice, a lack of resources, and a lack of experience with the medical environment are some of the many challenges nursing students have in the clinical context ( Panda et al., 2021). Li et al. (2022) assert that simulation-based education is a practical pedagogical approach for nursing students. A secure and regulated framework provides students an intense clinical setting for skill development and experiential learning ( Tamilselvan et al., 2023). This educational method fosters the development of several competences, including knowledge, skills, interprofessional collaboration, critical thinking, empathy, and a passion for learning ( Rattani, 2020, Tamilselvan et al., 2023). Furthermore, students can implement the concepts acquired in intricate and demanding scenarios ( Chernikova et al., 2020). A recent meta-analysis revealed that simulation enhances nursing students’ knowledge acquisition more effectively than computer-based simulation. In nursing education, the combination of simulation and computer-based simulation is the most prevalent blended learning methodology (student stressors).

Combining theoretical knowledge with practical skills is crucial for patient care, and simulation discussions promote students’ reflection, critical thinking, and reasoning ( Bailey & Emory, 2022).

The study results showed that participants believed simulation is about preparing for real life and realistic situations. The simulation sessions helped students handle emergency obstetric situations and prepared them for upcoming events ( Khan et al., 2023). The study also identified several new issues that will be considered, such as the gap between theoretical knowledge and practical abilities and the need for more practice time in simulation labs. Simulation-based learning methods are becoming increasingly popular in nursing education to mimic real-life conditions ( Somerville et al., 2023). They can offer an alternative to realistic and real-life situations in clinical encounters ( Leighton et al., 2021). Simulation helps students adapt to professional occupations and prepares them for real-life situations ( Mishra, Hemlata & Trivedi, 2023). Students in this study reported that simulation aided their skill development and knowledge transfer during neonatal resuscitation. These findings consistently show that simulation enhances practicing proficiency, skills, and confidence ( Alalhareth & Howarth, 2020; Mishra, Hemlata, & Trivedi, 2023). The study’s findings support the idea that simulation is an effective teaching strategy that can help students put theory into practice ( Wong & Kowitlawakul, 2020). In addition, during neonatal emergencies, such as breathing difficulties after birth, effective communication was enabled through calls, shouts for help, interactions, and role allocation—skills essential for successful interprofessional teamwork ( Khan et al., 2023). These scenarios’ actions are consistent with earlier research showing that simulation enhances students’ teamwork and communication skills ( Hustad et al., 2019).

### Limitation

It is important to recognize the limitations of the current investigation. This study was conducted at a single nursing school in the United Arab Emirates. The findings might not be as applicable to other nursing programs in this setting. Another limitation of this qualitative study was the small sample size, which might not accurately reflect the general population. Future research should therefore get beyond these restrictions by employing quantitative techniques, greater sample sizes, longer study durations, and other study locations.

## Conclusion and recommendation

The study findings indicate that male nursing students received the overall simulation experience well. However, it can be ameliorated by promoting active learning and integrating simulation-based learning with clinical practice. The study highlights the importance of skilled and encouraging simulation instructors who can provide participants with valuable guidance and feedback and the need for more individualized teaching. These results provide important new information about how simulation-based learning could improve nursing education and further studies in this field. Furthermore, the findings shed light on the problems that male nursing students have been dealing with for years and highlight efforts to make changes as soon as feasible. Based on the study results, it may be helpful to revise the simulation to provide more opportunities for independent problem-solving skills and to incorporate more real-life factors and variables into the scenario. Developing more individualized approaches to building self-confidence may be helpful, such as providing personalized feedback and guidance based on each student’s strengths and weaknesses. Incorporating more opportunities for reflection and self-assessment may also help build self-confidence.

## Data Availability

The data generated and analyzed during this study are not publicly available due to ethical and confidentiality considerations, as outlined by the Ethics Committee of the Office of the Vice Chancellor for Research & Graduate Studies at the University of Sharjah, UAE. Participant data contain sensitive personal information, and sharing such data publicly could compromise confidentiality and anonymity. The Institutional Review Board (IRB) has mandated that data sharing is permissible only under specific conditions that ensure participant privacy and align with ethical guidelines. Access to the data may be granted to qualified researchers for legitimate academic purposes upon request. Requests for access must be submitted in writing to the corresponding author Dr. Richard Mottershead rmottershead@sharjah.ac.ae. Applicants are required to provide a detailed research proposal outlining the purpose of their request and how the data will be used. Additionally, applicants must agree to adhere to strict confidentiality agreements and institutional guidelines regarding data handling. Access will be granted at the discretion of the Ethics Committee, subject to the signing of a data use agreement.
