# Supplementary material for: Exploring simulation as a teaching pedagogy for male undergraduate nursing students: A Qualitative Study in the United Arab Emirates
Source: F1000Res. 2025 Oct 6;14:920. Originally published 2025 Sep 15. [Version 2] doi: 10.12688/f1000research.167350.2 (PMC12583910; doi:10.12688/f1000research.167350.2)
Supplement: Peer review history

# Reviewer report: Reviewer response for version 2

**DOI:** 10.5256/f1000research.188736.r421782

**Published:** 2025-11-04

**Authors:** Mohamed BENFATAH

**Affiliations:** 1 Hassan First University of Settat, Settat, Morocco

**Summary:**

 This manuscript presents a descriptive qualitative study exploring the experiences of male undergraduate nursing students using simulation-based learning at the University of Sharjah, UAE. Forty-three students participated through individual interviews and two online focus groups. Thematic analysis revealed four main themes: (1) students’ perceptions of simulation, (2) preparation for realistic situations, (3) feelings of anxiety and stress, and (4) theory-practice gaps. The authors report that simulation enhances knowledge, practical skills, communication, self-confidence, and integration of theory into practice, while also revealing challenges such as anxiety, limited lab time, and gaps between theory and practice.

 Overall, the manuscript is well-written, comprehensive, and provides novel insights into the experiences of male nursing students in a culturally specific context. Simulation is positioned as a valuable pedagogical tool for bridging clinical training gaps.

**Evaluation of Key Aspects**

1. **Originality and Relevance:**
  - **Strengths:** The study addresses a clear gap in nursing education research, particularly the experiences of male students in maternity and neonatal simulation training in a culturally conservative environment. The topic is timely and relevant to educators seeking alternative methods to supplement clinical placements.
  - **Areas for Improvement:** None significant. The manuscript is original and contributes to the field.
2. **Methodology:**
  - **Strengths:** The study uses an appropriate qualitative design with semi-structured interviews and focus groups. Thematic analysis following Braun & Clarke’s six-phase approach is appropriate, and the use of NVivo 12 for coding adds rigor. Ethical considerations are well-addressed.
  - **Weaknesses / Points to Address:**
    - **Sampling Clarification:** The text mentions 32 participants in interviews but 43 in total; a clear table or figure summarizing participant distribution across interviews and focus groups would improve clarity.
    - **Interview Guide Transparency:** Although Table 1 includes questions, providing the full interview and focus group guide in a supplementary appendix would enhance reproducibility.
    - **Reflexivity Statement:** The authors do not discuss their positionality or potential biases as researchers, which is important in qualitative research to ensure credibility. A brief statement on the researchers’ background, relationship with participants, and steps taken to minimize bias should be added.
3. **Data Analysis and Rigor:**
  - **Strengths:** Thematic analysis is described in detail. Steps to resolve coding disagreements are explained, and triangulation with focus groups increases credibility. Data storage, confidentiality, and ethical considerations are thorough.
  - **Weaknesses / Points to Address:**
    - **Codebook and Theme Validation:** Consider including an anonymized excerpt of the codebook or examples of coding to illustrate transparency. This is optional but strengthens reproducibility.
    - **Data Saturation:** The authors mention that 25 participants are enough per Charmaz (2006). It would strengthen the manuscript to explicitly state how saturation was confirmed during analysis.
4. **Results:**
  - **Strengths:** The results are well-organized into four main themes with subthemes, supported by participant quotes. The findings align with the study objective and provide clear insight into students’ experiences.
  - **Weaknesses / Points to Address:**
    - Some quotes are repeated or paraphrased multiple times, which can be condensed for clarity.
    - Consider a table summarizing the themes, subthemes, and representative quotes; this improves readability for journal reviewers and readers.
5. **Discussion:**
  - **Strengths:** The discussion contextualizes findings within existing literature, highlighting the positive effects of simulation on skills, confidence, and communication. Cultural and gender-specific challenges are acknowledged.
  - **Weaknesses / Points to Address:**
    - Some parts of the discussion are repetitive, particularly when addressing stress, anxiety, and theory-practice gaps. Condensing and synthesizing these points will improve clarity.
    - Consider explicitly linking recommendations (e.g., increasing lab time, individualized feedback) to the literature and study findings in a concise table or paragraph.
6. **Limitations:**
  - Clearly stated (single institution, small sample).
  - Consider adding a comment on potential social desirability bias in interviews, especially as students were discussing sensitive experiences.
7. **Conclusion and Recommendations:**
  - Clear and actionable recommendations are provided.
  - Ensure the conclusion explicitly states how findings contribute to advancing nursing education, particularly for male students in culturally restricted contexts.
8. **References:**
  - Current references appear appropriate and recent. Ensure all references are correctly formatted according to the journal guidelines.

**Specific Points that Must Be Addressed to Ensure Scientific Soundness:**

 Issue Recommendation Participant distribution clarity: Provide a table showing total participants, interviews, and focus groups to remove ambiguity.

 Interview/FGD guide transparency: Include full interview and focus group guide as supplementary material.

 Reflexivity: Add a brief statement on researcher positionality, potential bias, and mitigation strategies.

 Saturation: Explicitly describe how data saturation was assessed.

 Coding transparency: Consider including a sample of coding or excerpts from the codebook.

 Repetitive quotes: Condense similar quotes to improve readability.

 Discussion clarity: Synthesize repetitive points on anxiety, stress, and theory-practice gaps; link recommendations directly to findings.

**Additional Constructive Suggestions:**

1. Adding a visual summary (e.g., table or figure) of themes and subthemes with representative quotes.
2. Consider highlighting gender- and culture-specific insights in a separate paragraph to strengthen the manuscript’s unique contribution.
3. Minor language edits for conciseness and clarity, particularly in Results and Discussion, will enhance readability.

**Overall Assessment:**

 This manuscript is scientifically sound, original, and relevant. With minor clarifications and revisions—particularly around methodology transparency, reflexivity, and condensation of repetitive text it will be suitable for indexing in journal.

Is the work clearly and accurately presented and does it cite the current literature?

Yes

If applicable, is the statistical analysis and its interpretation appropriate?

Yes

Are all the source data underlying the results available to ensure full reproducibility?

Yes

Is the study design appropriate and is the work technically sound?

Yes

Are the conclusions drawn adequately supported by the results?

Yes

Are sufficient details of methods and analysis provided to allow replication by others?

Yes

Reviewer Expertise:

Nursing education, simulation-based learning, clinical pedagogy, qualitative research methods in healthcare education.

I confirm that I have read this submission and believe that I have an appropriate level of expertise to confirm that it is of an acceptable scientific standard.

# Reviewer report: Reviewer response for version 2

**DOI:** 10.5256/f1000research.188736.r420842

**Published:** 2025-11-03

**Authors:** Agussalim Agussalim

**Affiliations:** 1 Makassar Health Polytechnic, Makassar, Indonesia

No further comments from my end

Is the work clearly and accurately presented and does it cite the current literature?

Yes

If applicable, is the statistical analysis and its interpretation appropriate?

Partly

Are all the source data underlying the results available to ensure full reproducibility?

No

Is the study design appropriate and is the work technically sound?

Yes

Are the conclusions drawn adequately supported by the results?

Yes

Are sufficient details of methods and analysis provided to allow replication by others?

Yes

Reviewer Expertise:

Nursing in Medical surgical Research

I confirm that I have read this submission and believe that I have an appropriate level of expertise to confirm that it is of an acceptable scientific standard.

# Reviewer report: Reviewer response for version 2

**DOI:** 10.5256/f1000research.188736.r421780

**Published:** 2025-11-03

**Authors:** Mulyadi Mulyadi

**Affiliations:** 1 Sam Ratulangi University, , Manado,, Indonesia

Thank you for the opportunity to review this manuscript, title: Exploring simulation as a teaching pedagogy for male undergraduate nursing students: A Qualitative Study in the United Arab Emirates

 I have provided the following feedback which I believe will strengthen the manuscript.

**Abstract:**

 The conclusion is meaningful but could be more focused on the main research theme. It would be helpful if the authors emphasized how their findings contribute to nursing education, especially in enhancing simulation learning experiences for male nursing students. Highlighting this connection would make the study’s message clearer and more impactful for readers.

**Methods:**

*The interview questions can be found in Table 1*. The interview guide is clear, but the authors should explain the theoretical or empirical basis used to develop it. This would strengthen the study’s methodological rigor and ensure alignment with the research objectives.

**Study rigor**

 The authors provide clear ethical safeguards for confidentiality and data security. To further strengthen this section, a brief explanation of how trustworthiness and methodological rigor, such as credibility, transferability, dependability, and confirmability, were ensured would enhance the study’s transparency and robustness

**Results and Discussion:**

- The results are clearly presented. However, mentioning the number of subthemes identified under each main theme would improve the clarity and completeness of the findings.
- The third theme, “Feelings of Anxiety and Stress,” lacks subthemes and is supported by only one participant quote (out of forty-three male students). While not all themes require subthemes, this limited evidence may not adequately capture the participants’ overall experiences. The authors are encouraged to provide additional supporting data or reconsider whether this theme should stand alone.

Is the work clearly and accurately presented and does it cite the current literature?

Yes

If applicable, is the statistical analysis and its interpretation appropriate?

Partly

Are all the source data underlying the results available to ensure full reproducibility?

Partly

Is the study design appropriate and is the work technically sound?

Yes

Are the conclusions drawn adequately supported by the results?

Yes

Are sufficient details of methods and analysis provided to allow replication by others?

Yes

Reviewer Expertise:

Nursing education, trauma and emergency nursing

I confirm that I have read this submission and believe that I have an appropriate level of expertise to confirm that it is of an acceptable scientific standard.

# Reviewer report: Reviewer response for version 1

**DOI:** 10.5256/f1000research.184457.r414848

**Published:** 2025-09-23

**Authors:** Agussalim Agussalim

**Affiliations:** 1 Makassar Health Polytechnic, Makassar, Indonesia

Summary of the Article

 This study, *“Exploring simulation as a teaching pedagogy for male undergraduate nursing students: A Qualitative Study in the United Arab Emirates”* (Dias et al., 2025), investigates the experiences of male nursing students with simulation-based learning in a context where clinical placements, especially in maternity care, are limited due to cultural barriers. Using a descriptive qualitative design, the authors conducted semi-structured interviews with 32 male nursing students and two focus groups (11 additional participants), for a total of 43 students. Data were analyzed using Braun and Clarke’s six-phase thematic analysis framework.

 Four themes were identified: (1) Students’ perceptions of simulation (e.g., improved skills, self-confidence, and communication), (2) Preparation for realistic situations, (3) Feelings of anxiety and stress, and (4) Theory–practice gaps. The authors conclude that simulation is a valuable and well-received pedagogy for male students, though improvements are needed in lab time, individualized feedback, and integration with clinical practice.

 Evaluation Against Review Questions

**1. Is the work clearly and accurately presented and does it cite the current literature?**

*Yes.* The manuscript is clearly structured (IMRaD), with transparent reporting of objectives, methods, results, and discussion. The literature cited is current, including very recent studies (2022–2025), and covers both global and regional perspectives. Some reliance on the authors’ own prior work is noted, but this does not overshadow the breadth of references.

**2. Is the study design appropriate and is the work technically sound?**

*Yes.* The descriptive qualitative design is entirely appropriate for the research aim of exploring experiences and perceptions. The sampling strategy, data collection methods, and analytic approach are sound. Rigor is addressed through triangulation, ethical safeguards, and anonymization. The main technical limitation is that the study is confined to one institution, which restricts transferability, but this is acknowledged.

**3. Are sufficient details of methods and analysis provided to allow replication by others?**

*Partly.* The methodology is generally well described, including sampling, data collection, and thematic analysis steps. However, some details are missing:

- The **interview and focus group guides** (or at least example questions) are not provided, making it difficult to replicate the data collection exactly.
- The **software or tools used for data analysis** are not specified (e.g., NVivo, Atlas.ti, or spreadsheets).
- It is not clear how **disagreements in coding** were handled (e.g., consensus meetings, inter-rater reliability checks).

**Recommendation:** The authors should include an appendix with the interview guide, clarify whether any qualitative analysis software was used, and describe how coder agreement was reached. This would significantly strengthen transparency and replicability.

**4. If applicable, is the statistical analysis and its interpretation appropriate?**

*Not applicable.* As a qualitative study, no statistical analysis is required. Thematic analysis is appropriate and correctly applied, with adequate use of participant quotations to support interpretation.

**5. Are all the source data underlying the results available to ensure full reproducibility?**

*Partly.* The raw interview and FGD transcripts are not publicly available due to confidentiality concerns, but the authors provide a **controlled access pathway** for qualified researchers via ethics approval. This is standard practice in qualitative research involving sensitive personal data.

**Recommendation:** The current data availability statement is acceptable, but the authors could improve transparency by providing **an anonymized sample transcript or coding framework** in supplementary material. This would give readers a clearer sense of the raw data without breaching confidentiality.

**6. Are the conclusions drawn adequately supported by the results?**

*Yes.* The conclusions align well with the identified themes and are supported by direct quotations from participants. Recommendations (e.g., individualized feedback, integration of simulation with clinical practice) logically follow from the findings. The limitations are acknowledged, which keeps the conclusions appropriately cautious.

 Overall Strengths

- Timely and relevant focus on male nursing students in a cultural context with clinical placement restrictions.
- Clear and logical structure with transparent reporting of ethical considerations.
- Use of triangulation (interviews + FGDs) and thematic analysis strengthens trustworthiness.
- Engagement with very recent literature makes the study well-situated in current debates.

Overall Limitations & Required Revisions

- **Interview/FGD guide not included** → Must be added (appendix or supplementary file).
- **Analysis software not specified and coder agreement process unclear** → Should be clarified.
- **Data availability** → While full transcripts cannot be shared, providing an anonymized sample transcript or coding framework would strengthen reproducibility.

Final Recommendation

 The manuscript is scientifically sound, clearly presented, and contributes valuable insights into simulation-based pedagogy for male nursing students. However, **minor revisions are required** to strengthen methodological transparency and reproducibility:

1. Include interview/FGD guide questions (appendix/supplement).
2. Clarify analytic tools used and how coding disagreements were resolved.
3. Consider sharing an anonymized transcript excerpt or coding framework to enhance reproducibility.

With these revisions, the paper will meet high scientific and reporting standards.

Is the work clearly and accurately presented and does it cite the current literature?

Yes

If applicable, is the statistical analysis and its interpretation appropriate?

Partly

Are all the source data underlying the results available to ensure full reproducibility?

No

Is the study design appropriate and is the work technically sound?

Yes

Are the conclusions drawn adequately supported by the results?

Yes

Are sufficient details of methods and analysis provided to allow replication by others?

Yes

Reviewer Expertise:

Nursing in Medical surgical Research

I confirm that I have read this submission and believe that I have an appropriate level of expertise to confirm that it is of an acceptable scientific standard, however I have significant reservations, as outlined above.

# Response

**Published:** 2025-09-26

**Authors:** Dr. Richard Mottershead

**Affiliations:** College of Health Sciences, University of Sharjah, Sharjah, Sharjah, United Arab Emirates

Dear editor and reviewers,

 Thank you for your email and comments on our manuscript. We revised our manuscript in

 response to the reviewers' comments and concerns. Our revisions and comments are highlighted in yellow throughout the manuscript. Please see our responses below.

 Reviewer Comments:

 The manuscript is scientifically sound, clearly presented, and contributes valuable insights into

 simulation-based pedagogy for male nursing students. However, minor revisions are required to strengthen methodological transparency and reproducibility:

 1. Include interview/FGD guide questions (appendix/supplement).

 Response: Thank you so much for your comment. We added and included the interview guide

 questions in the appendix.

 1. How would you describe your overall experience during the maternity practicum labs?

 ( Probe: What stood out the most to you?)

 2. What were your feelings and thoughts on the first day of your maternity practicum?

 (Probe: Were you nervous, excited, or unsure?)

 3. How did your feelings change by the last day of the practicum?

 (Probe: What had improved or become more comfortable?)

 4. Can you share a positive experience you had during your maternity practicum?

 (Probe: What made it a good experience?)

 5. Can you describe a negative or challenging experience during the practicum?

 (Probe: How did you handle it?)

 6. What was the most difficult part of your experience in the maternity lab or clinical setting?

 (Probe: Skills, communication, environment, etc.)

 7. As this was your first exposure to maternity care, what specific fears or anxieties did you

 experience?

 (Probe: Were any of these related to gender, confidence, or knowledge?)

 8. Did participating in the maternity practicum affect your daily life or your mental/emotional

 health? If so, how?

 9. What specific challenges did you face as a male student in the maternity practicum setting?

 (Probe: Interaction with patients, acceptance by staff, cultural barriers, etc.)

 10. What advice would you give to a second-year male student who will soon enter the maternity

 practicum?

 (Probe: What do you wish you had known beforehand?)

 11. How were you able to apply maternity theory learned in class during your practicum or

 simulation sessions?

 (Probe: Can you give specific examples?)

 12. Would you consider working in the maternity department in the future? Why or why not?

 (Probe: Did your practicum influence this decision?)

 13. How did simulation activities in the lab prepare you for real-life maternity clinical experiences?

 (Probe: What scenarios or skills were most helpful?)

 2. Clarify the analytic tools used and how coding disagreements were resolved.

 Response: Thank you for your comment. This study employed a thematic analysis, adhering to the six data analysis phases described by Braun and Clarke (2006). We employed NVivo 12 as our data management tool, which assists us in coding and identifying themes— thematic analysis. These tool helps us interpret interviews and focus groups, uncover patterns, and derive insights in identifying themes. (highlighted in yellow).

 Coding disagreements in qualitative research can happen when different coders assign varying labels to the same data, reflecting different interpretations or ambiguities. This study addresses coding disagreements through systematic procedures, including detailed discussions where coders justify their choices, consult established guidelines, and seek input from third parties to maintain transparency and clarity. During our analysis, the authors meet to review all coding and reach consensus. Regular meetings are held throughout the process to review the data and implement any necessary adjustments.

 3. Consider sharing an anonymized transcript excerpt or coding framework to enhance

 reproducibility.

 Response: Thank you very much for your comment. In this study, reproducibility is achieved by obtaining consistent results through the thematic analysis steps, coding, and conditions. We provided clear, specific, and thorough information about the data products that support our

 published results, allowing other researchers to replicate the analysis. Additionally, all data are

 available from the first author or the corresponding author upon reasonable request.

 4. Are all the source data underlying the results available to ensure full reproducibility?

 Response: Thank you. Participant data contains sensitive personal information, and sharing such data publicly could compromise confidentiality and anonymity.

 The Institutional Review Board (IRB) has mandated that data sharing is permissible only under specific conditions that ensure participant privacy and align with ethical guidelines. Access to the data may be granted to qualified researchers for legitimate academic purposes upon request.

 Requests for access must be submitted in writing to the corresponding author, Dr. Richard

 Mottershead.
